# Challenge-Based Learning in Dental Education

**DOI:** 10.3390/dj11010014

**Published:** 2023-01-03

**Authors:** Mohammed Zahedul Islam Nizami, Vicky Wenqing Xue, Amy Wai Yee Wong, Ollie Yiru Yu, Conson Yeung, Chun Hung Chu

**Affiliations:** Faculty of Dentistry, University of Hong Kong, Hong Kong SAR 999077, China

**Keywords:** dental education, challenge-based learning, problem-based learning

## Abstract

Challenge-based learning (CBL) is a novel learning framework for a collaborative and multidisciplinary learning experience. It allows students, teachers, stakeholders, researchers, families, and society to work together to identify and solve real-world challenges. CBL helps students develop a deeper knowledge of the subjects they are studying. The concepts of CBL originate from a variety of educational theories and approaches, such as problem-based learning and inquiry-based learning. The precursor to the CBL framework is problem-based learning. However, unlike in problem-based learning and other approaches, students formulate the challenges they will address in CBL. Furthermore, students need to create a solution resulting in concrete action. CBL takes into account the social impact of an idea rather than just the corporate benefits. Therefore, it can help students expand the scope and depth of learning, encourage teamwork capabilities, and raise their awareness about considering quality and ethics in decision-making. CBL is implemented in universities, schools, and institutions worldwide and its use is well-recognized in science, engineering, and medicine, but it has not been translated into dentistry. The aim of this paper is to describe the concept of inclusion, principles and design, implementation, and supervision of the CBL framework in a dental course for the adaption of this learning framework to dental education.

## 1. Introduction

Today’s students have instant access to information by using the web and informal learning. For this reason, the conventional ‘chalk and talk method’ of teaching is becoming less effective in engaging students in learning [1] and motivating them to achieve their goals [2]. These days, students are presented with content-centred coursework. This meets certain standards but lacks real-world settings and opportunities for active participation. An accessible, effective, and efficient framework to solve these problems is necessary. Challenge-based learning (CBL) was first developed by the technology company Apple, and it is now implemented in universities, schools, and institutions worldwide, as an advanced solution for educational learning systems [2,3,4].

Different studies emphasized the importance of active learning tools, which prepare students for lifelong, self-regulated, and cooperative learning; at the same time, these learning tools provide high-quality learning, according to students’ metacognitive and self-regulatory skills. From this perspective, Challenge-Based Learning (CBL) is a pedagogical approach, with roots in experimental learning, where the starting point for learning is an open-ended, real-life challenge [5]. CBL is an engaging multidisciplinary approach to teaching and learning. It encourages students to use current technology to solve real-world problems. Traditional teaching methods focus on imparting highly specific and methodical knowledge through teacher-centred didactic teaching (i.e., lectures, reading books, classroom-based activities, and the repetition of work) to help students gain a growing knowledge of the content [6]. In contrast, the new educational strategy allows students to be exposed to real-life situations where they face high levels of uncertainty and challenges. Furthermore, the new technologies, currently available, allow the identification of the preferred learning style of modern students and, at the same time, enhance the diversification of the teaching style [7]. The new model, based on skill development, develops a transition from a face-to-face classroom setting to an educational setting in the real world [8].

The concepts of CBL were derived from a variety of educational theories and pedagogical approaches, including problem-based learning (PBL), inquiry-based learning (IBL), and the conceive, design, implement, operate method (CDIO). PBL is considered the main precursor to the CBL framework. PBL has been widely applied in medicine and engineering education because it can improve critical thinking, self-guided learning, generic skills, and the long-term retention of students [9,10]. The ‘ASK’ (attitude, skills, knowledge) model distinguishes PBL from the traditional teaching method [11]. In PBL, a group of students are presented with a design, research, or diagnostic problem. Learning takes place through the process of finding a solution [12,13]. IBL is defined in different ways in the literature. Generally, it is a student-centred approach. In IBL, the instructor guides the students through questions they pose, methods they design, and data they interpret. The concept involves intensive questions on knowledge creation and problem-driven student projects [14]. IBL can be effective in enhancing students’ ability to search, research, and solve problems. However, it can also be perceived as a risk-taking process because it may result in lower satisfaction with students’ assessments [15]. The CDIO method is specifically used in engineering education. It is an educational framework that emphasizes engineering fundamentals in the context of imagining, designing, executing, and operating real-world systems and products [16,17].

Unlike the above mentioned learning frameworks, CBL requires students to formulate the challenges they will address. Furthermore, CBL is a multidisciplinary approach and engages teams composed of learners (students), facilitators (teachers), stakeholders, families, and society. CBL considers the social impact of an idea rather than just the corporate benefits. Therefore, CBL can expand the scope and depth of learning and encourage teamwork capabilities as well as personal awareness in students through considering quality and ethics in decision-making. In the process of designing scholarly and pedagogical education systems (CBL) for the next generation, as a learning group, we need to critique the systems we are developing and examine our theories and practices. At the same time, we need to reveal how the other parts of an education system interrelate and how this system works within the context of whole existing education systems [18].

It is now time to advance dental education via the CBL framework, as it is a truly student-centred community-based learning method for handling real-world situations. Medical and nursing schools have already implemented CBL in their course design [19,20,21,22]. In dentistry, CBL has not yet been implemented. Therefore, the aim of this study is to provide an overview of a course design for implementing CBL in dentistry to provide best practices, solve frequently asked questions, and adapt the learning approach to dental education.

## 2. CBL in the Education System

CBL is a collaborative and hands-on approach. A true student-centred education system requires students to work with their peers, teachers, communities, and experts worldwide to develop in-depth knowledge by identifying challenges and sharing their results with the world. Research has shown that student-centred learning approaches are effective in improving students’ learning [23,24]. The first outline of CBL was published in a white paper in 2008 [3]. Since then, academicians and schools around the world have gradually accepted and applied it to improve teaching and learning, allowing students to achieve distinction quickly in their communities. Later, in 2009, a detailed study design was published by the New Media Consortium for classroom practice. The study recruited six schools involving twenty-nine teachers and three hundred thirty students in seventeen disciplines. The study found that CBL is effective in promoting learning [24]. Furthermore, in 2011, the study was expanded to nineteen schools involving ninety teachers and one thousand five hundred students from three different countries and demonstrated that this pedagogical approach is an excellent way to engage students, meet curriculum standards, and achieve twenty-first-century skills. In addition, CBL was suggested for students of all age groups [4].

It has been reported that adopting this framework requires a profound transformation of the organizational culture. This involves teacher training, rebuilding infrastructure, and transforming it into an administrative framework that is both open and flexible to change [25,26]. The challenge method is not difficult to work with because most students are familiar with the concept, as they have watched multiple reality shows on television. The common theme is that competitors are presented with a challenge that requires them to use their creativity to draw on previous learnings, gain new knowledge, work as a team, and reach solutions. This idea is successful because participants are highly motivated by the general goal of winning an award afterward [27].

**Figure 1:**
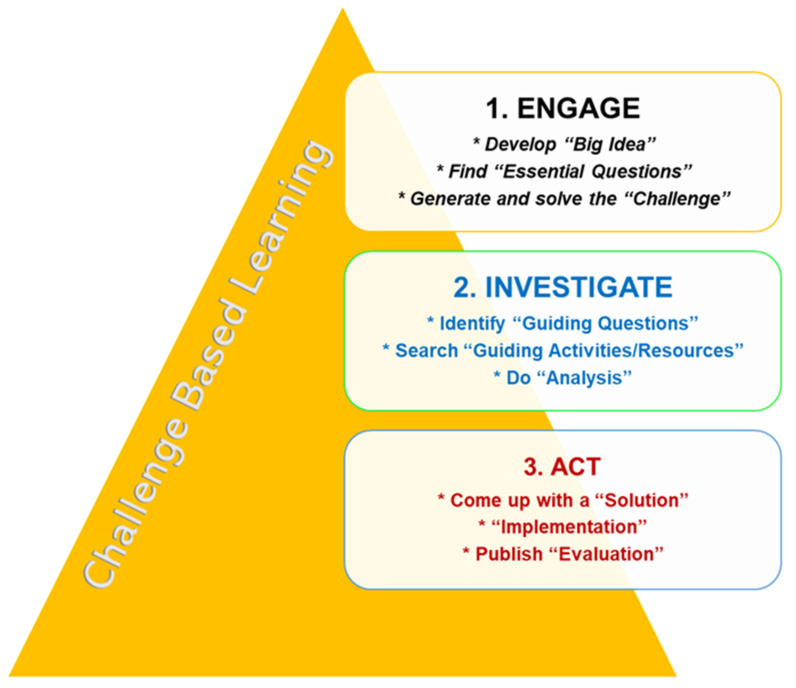
The three phases of CBL (adapted from Nichols et al., 2016) [2].

The CBL framework has been expanded to some new areas such as strategic planning and training in the workplace [28] and mobile software instruction and development [29]. Most recently, in 2016, the ‘Digital Promise’ team and the founding CBL team members jointly updated the CBL content. CBL was applied to the collaboration project ‘Apple Classrooms of Tomorrow’ with great success among public middle schools, universities, and research organizations [30,31]. They developed a website and published a book [2]. CBL is presented in three phases (Figure 1).

The first phase is ‘Engage’. Through a process of essential questioning, learners (students) move from a big idea to a concrete and effective challenge. The second phase is ‘Investigate’. All students plan and take part in a journey that forms the basis of solutions and addresses academic requirements. The third phase is ‘Act’. Evidence-based solutions are created for application to authentic audiences. They will be evaluated based on the results.

## 3. Principles of CBL

CBL is a flexible framework that implements new concepts and study designs and generates new study models [2,3,4]. The following twelve items (a through l) are the basic principles of CBL:

(a) A flexible and customizable framework that can be applied as a guiding pedagogy or integrated with other progressive methods of learning;
(b) A walkable model with multiple points of entry and the capability to start small and create big;
(c) An open framework without any proprietary concept, product, or subscription;
(d) A process that puts all students in charge and manages learning;
(e) An authentic environment to meet academic standards and establish a deep connection with the content;
(f) A focus on global ideas, meaningful challenges, and the development of local and age-appropriate solutions;
(g) An authentic relationship between academic disciplines and real-world experience;
(h) A framework for developing twenty-first-century skills;
(i) The purposeful use of technology for researching, analysing, organizing, collaborating, networking, communicating, publishing, and reflecting;
(j) The opportunity for students to make a difference;
(k) A way to document and assess both the learning process and products;
(l) An environment for deep reflection on teaching and learning.

## 4. Design of CBL

Many of today’s problems cannot be solved with just one discipline. These problems require a multidisciplinary approach because solutions require technical knowledge, social perspectives, and communal understanding. In CBL, learners (students), facilitators (teachers), stakeholders, families, and society play different roles [2,3,4].

### 4.1. Role of the Learners (Students) in CBL

In CBL design, students are not passive learners. It is not like the traditional student-teacher role. They work together with facilitators, researchers, entrepreneurs, and external agencies (stakeholders) to explore and address current challenges.

Apprenticeship teams are usually formed by students from various backgrounds, such as different study levels and programs.

### 4.2. Role of the Facilitator (Teacher) in CBL

The role of the teacher is to be a facilitator for students, to help them with guidance, knowledge, and real-world information. The teacher provides relevant content skills. The teacher is an instructor in the student team, supporting them at various stages of the CBL and facilitating their learning. Teachers can play a role in defining learning outcomes at the course level. However, the contents of these lessons can be flexible according to individual students’ preferences.

### 4.3. Role of the Stakeholder in CBL

Sometimes, especially in community-involved learning, educating students with close guidance from stakeholders is important for their practical life and future engagement. In some courses, students will have to invite community stakeholders as guest lecturers or to be a part of their real-life projects. The stakeholder will guide the process. They will also get active feedback from the students. In ideal situations, they are partners of the students. Therefore, students should inform stakeholders about the pros and cons involved in the project when the students are trying to solve issues.

### 4.4. Role of Communities in CBL

In CBL, students face real-world challenges; thus, the involvement of all stakeholders and community members of society can effectively impact the investigation of the problem through their active participation in its actual solution.

### 4.5. Implementation of CBL

Each educational design is planned to follow a systematic process. Intellectually created educational designs have been proposed and implemented in the process. CBL has been adopted from some existing education systems. For this reason, we can easily implement it in our education system.

### 4.6. Redesigning Existing Courses

CBL can be included in existing courses. The challenge can be translated into a real-life application in accordance with the course. When incorporating challenges into existing courses, it is important to keep learning goals flexible. This is because students will make their own choices and manage their own learning. Teachers will not only be experts in the content of the course but will also be facilitators when students work on their challenges.

### 4.7. Extracurricular Learning Experiences

Extracurricular learning can also be applied in CBL. Students or outsiders will also focus on ‘big ideas’. The students then continue to do extracurricular activities along with other steps in the CBL curriculum to achieve good support. An experienced teacher can be a good trainer. However, the objects and strategies of learning will be defined by students in extracurricular learning. This will be beneficial when it is time to evaluate and earn credit.

## 5. CBL in Dental Education

Active learning strategies can engage students in a coherent discussion, causing them to comprehensively analyse situations and practice critical thinking. It can maximize the learning effect of the subjects on students [32]. PBL as an active learning method is widely used in medical and dental education. In PBL, real problems are used to trigger questions and create a learning setting for motivating students to be actively involved and to think critically [33]. This boosts students’ confidence. CBL is speculated to be a derivative of the PBL approach Therefore, it has quite similar characteristics, but it is different from PBL.

Those who are familiar with the PBL approach will be able to adapt and implement CBL in dental education more easily. Furthermore, CBL itself will drive the learning strategies by getting the entire learning team involved. Thus, CBL can be easily adapted and implemented in dental education and develop knowledge and skills through identifying problems in real-world situations and engaging communities with their challenges. Table 1 highlights some comparative characteristics of PBL and CBL.

The key point of CBL is that learning is driven by challenges that have more than a few solutions [2,3,4]. Unlike a more traditional curriculum, the design of CBL curriculums is similar to that of PBL curriculums. However, special attention should be paid to the mentality of the education team. In addition, it should be borne in mind that moving towards a new strategy (CBL) mindset itself is a big challenge. CBL leads students to be dynamic and complex thinkers and to stand out in unpredictable future tasks. In CBL, students work on multidisciplinary teams with challenges. It will improve their team skills and teach them how to solve problems and design solutions. Therefore, they develop their self-steering and learning skills. Additionally, CBL brings together interdisciplinary and disciplinary perspectives on problems and solutions related to environmental, social, health, education, and economic sustainability. Table 2 shows the CBL topics and descriptions. Appendix A is an example of CBL study design in dental education.

Today’s globalization, with its knowledge-based economy, creates a growing need for individuals to practice creative thinking skills. Creative thinking has become relevant in many advanced fields such as education, art, medicine, information and communications technology, and social media. Recent evidence has shown that creative thinking skills can be taught to improve problem-solving skills [40,41,42]. In this context, CBL has appeared as a quite new approach for engaging students in creative and advanced learning [24]. CBL incorporates modern technology, teamwork, self-learning, peer learning, and real-world problem-solving in its teaching tools. The learning process can be extended from the classroom to the local or global community. CBL has been found to develop students’ ability to learn by deepening their understanding of the material and extending their practical skills and engagement [4,24,31]. CBL has also been found to help learners perform better in group interaction, integration, and synthesis of concepts [28]. It has attracted academics through its immense learning outcomes [27,43].

In CBL, students are expected to ask questions, search the literature, conduct a primary survey, consult experts, and attempt to answer the essential questions hands-on. Based on the results, they will find challenging problems and may reframe the essential questions or guiding questions. Then, they will spend more time and effort generating innovative but realistic solutions for the problem, with an outline of the details of their resources and activities to develop the answer. In this approach, students will explore the ‘big ideas’ provided and creatively try to analyse and uncover the problems. However, in the beginning, it can be difficult to implement. Thus, facilitators may modify the strategic plan to avoid falling into the wrong path in learning. We believe this will help enhance students’ ability to innovate and learn. They will be good at generating new ideas and getting out of old thoughts. Logically, they will be able to share their creativity and innovation in useful and practical dental fields like disease management, dental product selection and innovation, best services, or related new program development. To date, we cannot find any published literature on the application of CBL in dental education. We will be using CBL in dental education in our institute and will observe the result. We certainly believe that this will be a great teaching approach. With the increase in life expectancy, promoting sound oral health is a global obligation. Therefore, the new dental student must be ready to deal with real-world challenges.

## 6. Conclusions

In conclusion, CBL is a collaborative and multidisciplinary learning experience for identifying and solving real-world challenges. It can be applied to dental education to allow dental students to create challenging questions from ‘big ideas’ and solve them based on a logical practical point of view. CBL enables students to gather knowledge from different sources and create a framework so that they can work together as a team to plan solutions to address the challenges. CBL can improve students’ general knowledge about different various specialties and products, the application of technologies, community involvement, and the management and treatment of orofacial diseases. CBL enables students to meet the real-world challenges of dentistry.

## Figures and Tables

**Table 1:** Learning methods of problem-based learning and challenge-based learning.

Topics	Problem-Based Learning	Challenge-Based Learning
**Learning**	Students learn the latest information by self-learning using designed problems. The knowledge is used to solve the problem at hand [34].	Students develop a deep knowledge of the subjects they are studying. The challenge itself triggers new knowledge generation and necessary tools or resources [24].
**Focus**	Students are confronted with a relevant problematic situation, which is often imaginary and does not require a real solution [35].	Students are confronted with an open, relevant, problematic situation that requires a practical solution [2].
**Product**	Students focus more on the learning processes than the products of the solutions [36].	Students need to create a solution resulting in concrete action [2].
**Process**	Students work with the problem in a way that tests their ability to reason and apply their knowledge to evaluate them according to their level of learning [37].	Students analyse, design, develop, and perform the best solution to deal with the challenge for them and other people to evaluate [4].
**Teachers’ role**	Facilitator, guide, tutor, or professional adviser [38].	Coach, co-researcher, and designer [39].

**Table 2:** CBL topics and description (adapted from Nichols et al., 2016) [2].

Topics	Description
**Big idea**	A broad concept that can be explored in more than one way. It critically engages students in society.
**Essential question**	The process of personalizing and finding important ideas in the ‘big idea’.
**Challenges**	A call to action is designed by teachers and students to create concrete actions as a solution.
**Guiding questions**	The learner community develops questions and finds and lists the knowledge and skills needed to develop an effective solution.
**Guiding activities and resources**	Activities that the students participate in, as well as the resources that students identify, are used to answer guiding questions.
**Analysis**	Process for exploring the answers to the guiding questions and identifying overarching themes and concepts. This lays the foundation for solutions.
**Solution: Implementation**	Concrete, effective, and descriptive concept to solve the challenge. Solutions are executed with an authentic audience.
**Evaluation**	Students evaluate their activities through the refined solutions and implementation of results.
**Publishing student solution**	Students compile their experiences and reflections, including the challenge description, learning processes, solutions, and implementation outcomes.
**Publishing student reflection**	Documents can be shared with the world through web-based communities. It is also good practice to hold a public event with all the participants.

## Data Availability

Not applicable.

## Appendix A

An example of CBL study design in dental education

**Big idea:** Dental caries

**Essential questions:** What is a caries-free healthy life?

**Challenge:** Caries prevention

***Big idea***—Suppose the team starts with a topic, ‘dental caries,’ which has broad meaning and importance to dentists, patients, dental educators, students, stakeholders, and society.

***Essential questions***—After developing a topic, the team will come up with some essential questions that reflect the interest in learning and the needs of society.

- *Questions will be developed by the students*—The ideas will define the topics according to the aetiology, prevention, and management of dental caries and the consequences and social impacts, including to general health and the economy, caused by caries.
- *How does the question emphasize the learning outcome?*—Depending on the developed questions, students will start thinking. They will go to the facilitator, stakeholders, or the public, involving both internal and external elements of the team to develop guidelines and solutions.

The topic ‘dental caries’ can be stated with the essential question: What is a caries-free healthy life?

*Challenge*—For every essential question, a challenge will be created to ask students to come up with a specific answer or solution. If we think about caries, the challenge is ‘caries prevention’. Students may come up with challenges related to:

- Development and prevention of caries;
- Effects on health and the economy related to caries.

***Guiding questions***—Students will generate questions to which they need to discover solutions to meet the challenges. Some guiding questions may be:

- What is dental caries?
- How are people affected by dental caries?
- How do people get protection against dental caries?
- How can mass education reach society, and how can dentists give the solution?
- What is the prevalence of caries in different ethnic groups, age groups, genders, etc.?
- What treatment strategies are available to solve the problem?
- What occurs in the absence of treatment?
- What are the strategies for configuring protection and prevention to serve society?
- How can stakeholders and the community be involved to serve society?
- How do dentists educate and create awareness in society?
- How do we find a realistic solution for treatment?

***Guiding activities***—Students will hold weekly discussions with facilitators, learn basic cariology from websites, lectures, seminars, and group discussions, and organize a discussion with the community (patients and dentists), social welfare officers, authorities from companies that make dental products and devices, etc. To achieve practical knowledge, students will attend university health clinics, local clinics, hospitals, or other dental schools and other classes.

***Guiding resources***—Students will use their research books, class lecture notes, literature, the internet, and expert opinions to develop solutions to their guiding questions. Videos on the internet will help them to learn how different communities and health and social workers applied preventive and restorative strategies.

***Solutions***—Students will create situation-specific solutions as well as general solutions during their evaluation. The critical aspects of data collection and management will be itemized and worked on by the entire group. Students will learn how to acquire the knowledge needed to solve each question, verify event logs for possible break-ins, and update existing treatments and applications. The group discussion on management will come up with a solution to prevent dental caries through enhancing social awareness and applying dental treatment. In general, they will have more than one solution to a problem. The solutions will be determined by the latest and most advanced knowledge, and they will be openly discussed.

***Study assessment and result publication***—Students will be evaluated based on their activities for implementing their knowledge to solve real challenges. They can compile records of experiences and results on a digital platform (e.g., audio, video, and photography; Word files; or PowerPoint presentations). It should be borne in mind that they must record their reflections with descriptions of the challenges, learning processes, solutions, and implementation outcomes. Finally, these results can be shared with the world through web-based communities. In addition, the arrangement of a public event to publish their results will boost their leadership skills.
